# Dietary intakes of branch chained amino acids and obesity risk among Chinese gestational diabetes women

**DOI:** 10.3389/fnut.2024.1436450

**Published:** 2024-10-10

**Authors:** Xiaoyun Yang, Weiqin Li, Wei Li, Huikun Liu, Leishen Wang, Junhong Leng, Yuxin Fan, Xilin Yang, Ming Liu, Gang Hu

**Affiliations:** ^1^Department of Endocrinology and Metabolism, Tianjin Medical University General Hospital, Tianjin, China; ^2^Pennington Biomedical Research Center, Baton Rouge, LA, United States; ^3^Tianjin Women’s and Children’s Health Center, Tianjin, China; ^4^Department of Epidemiology, School of Public Health, Tianjin Medical University, Tianjin, China

**Keywords:** branched chain amino acids, obesity, gestational diabetes mellitus, postpartum, overweight

## Abstract

**Introduction:**

Epidemiological studies have assessed the correlation between daily dietary branch chain amino acid (BCAA) intakes and the risk of obesity, however, the findings from these studies were inconsistent and investigations among GDM women were few.

**Objective:**

The present study was to investigate the associations of daily BCAA intakes with the risks of overweight and abdominal obesity among women with prior gestational diabetes mellitus (GDM) postpartum.

**Method:**

We performed a cross-sectional study of 1,263 women with prior GDM at 1–5 years post-delivery. Logistic regression models were used to estimate the associations of daily dietary intakes of BCAAs with the risks of overweight and abdominal obesity.

**Results:**

The multivariable-adjusted odds ratios (ORs) across quartiles of daily BCAA intakes postpartum were 1.42 (95% confidence interval [CI] 1.02–1.97), 1.00 (reference), 1.21 (95% CI 0.88–1.68), and 1.31 (95% CI 0.95–1.81) for general overweight, and 1.38 (95% CI 0.99–1.90), 1.00, 1.19 (95% CI 0.86–1.64), and 1.43 (95% CI 1.04–1.98) for abdominal obesity, respectively. Women with the lowest quartile of daily BCAA intakes significantly increased the risks of general overweight (OR 1.49; 95 %CI 1.06–2.09) and abdominal obesity (OR 1.50; 95 %CI 1.08–2.11) compared with women at quartile 2 of daily BCAA intakes after further adjustment of daily energy intake.

**Conclusion:**

The present study indicated that daily lower BCAA intakes were associated with increased risks of general overweight and abdominal obesity among women with prior GDM.

## Introduction

Gestational diabetes mellitus (GDM) is a serious complication during pregnancy (1), and has clinical impacts on maternal and offspring’s health during the perinatal and postpartum periods (2, 3). Approximately 12.8% of all pregnant women are diagnosed with GDM worldwide (4). A recent study has shown that about 14.8% of women are GDM in mainland China (5). A recent large meta-analysis and systematic review has shown that women diagnosed with GDM have a 10-fold increased risk to develop type 2 diabetes later in life (6).

Branched chain amino acids (BCAAs) including leucine, isoleucine and valine are nutritionally essential, which make up for 20% of the total protein content (7). The main physiological function of BCAAs is to promote protein synthesis of muscle, liver and adipose tissue, and they also participate in the tricarboxylic acid cycle and provide energy to cells. Based on the function of BCAAs in metabolism, many studies investigated the influence of BCAAs on metabolic diseases including obesity and insulin resistance. Some investigations focused on serum BCAAs and found that high serum BCAA levels might increase the obesity risk (8–10). BCAAs cannot be synthesized in humans and diet is their only source. The relationship between serum BCAA levels and BCAA intakes is not consistent among different studies (11, 12). Therefore, exploring the influence of daily BCAA intakes on metabolic health is very practically significant for nutrition intervention in daily life. There are some human studies investigating the associations between dietary intakes of BCAAs and the obesity risk, but the results are inconsistent (13–16).

Most of GDM women are overweight and obese after delivery (17), which could further increase the risk of diabetes among the higher risk individuals (18). As we all know, most women during the postpartum period would increase nutrition intake to recover the physical fitness, and the phenomenon is more significant among Chinese women because of the Chinese tradition. For these reasons, better weight management is more meaningful among GDM women postpartum, especially for Chinese women. However, to our knowledge, no studies have assessed whether postpartum BCAA intakes would influence the obesity risk in GDM women. The present study aimed to investigate whether daily dietary intakes of BCAAs after delivery were associated with postpartum overweight and obesity risks among women with GDM.

## Materials and methods

### Study population

**Figure 1:**
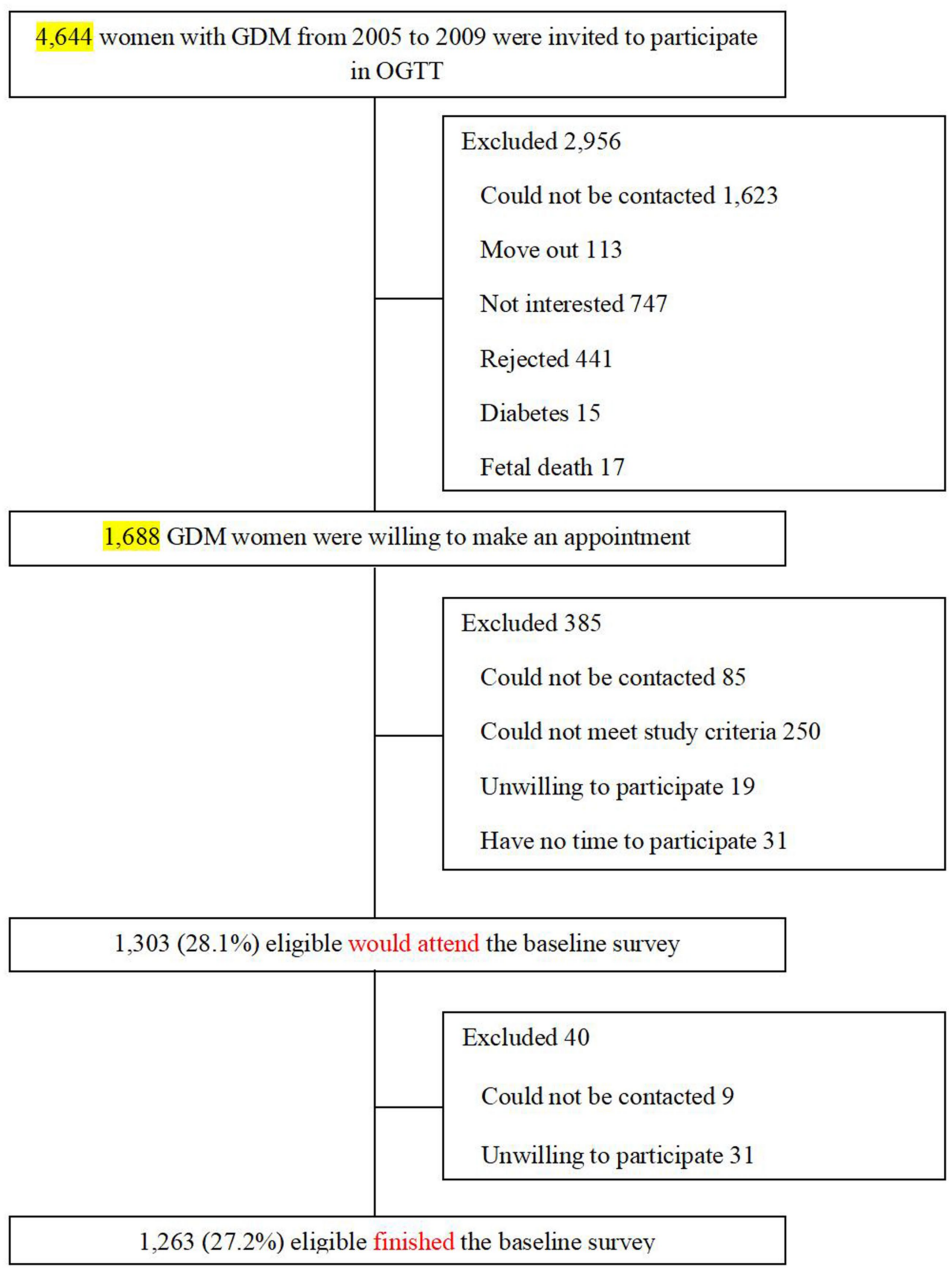
Participant flow chart.

The present analysis used the baseline survey data derived from the Tianjin GDM Prevention Program in China (19). Since 1999, all pregnant women living in six urban districts in Tianjin have participated in universal screening for GDM, and the average proportion of screened pregnancies was >91% from 1999 to 2008. A total of 128,125 pregnant women participated in the GDM screening program, and 6,247 were diagnosed with GDM. The diagnosis of GDM is based on the criteria set by the 1999 World Health Organization (WHO) (20). We chose all GDM women (*N* = 4,644) from the Tianjin GDM Prevention Program between 2005 and 2009, who participated in the screening visit. For the present study, the inclusion criterion was: age 20–49 years at the baseline survey. The exclusion criteria were: (1) age <20 or ≥ 50 years; (2) presence of any chronic diseases that could seriously reduce the life expectancy; (3) unable or unwilling to participate in the study or lose contact; (4) currently pregnant, or planning to become pregnant in the next 2 years. Between August 2009 and July 2011, 1,263 women with GDM finished the baseline survey at 1–5 years postpartum (19, 21). The participant rate is 27.2% (Figure 1). There were no differences in the OGTT at 26–30 weeks’ gestation between the returned and unreturned women with GDM, with regard to age (28.9 vs. 28.7 years), fasting glucose (5.34 vs. 5.34 mmol/L), 2 h glucose (9.23 vs. 9.16 mmol/L), and the prevalence of IGT (90.9% vs. 91.8%) and diabetes (9.1% vs. 8.2%). The study was approved by the Human Subjects Committee of the Tianjin Women’s and Children’s Health Center (Approval numbers: 2009-01, 2013-03-01 and 2017-03-01), and all participants provided written informed consent.

### Measurements

At the baseline survey, all participants completed a self-administered questionnaire, a dietary intake assessment and a physical examination. A self-administered questionnaire included sociodemographic characteristics, family history of diabetes, education level (<13 years of high school or less, ≥13 to <16 years of undergraduate education, ≥16 years with graduate education or above) and lifestyle questions (smoking habits, passive smoking status, alcohol status, and leisure time physical activity).

The methods used for assessing dietary intakes were food weighting plus three continuous days (two weekdays and one weekend day) food records. Standardized weighing scales were used for weighing the foods separately before being cooked or the portion sizes were estimated by participants. After completion, a dietitian reviewed and coded the records. Intakes of energy and nutrients including BCAAs (including leucine, isoleucine, and valine) were analyzed using the 2002 Chinese food composition tables (22). The food weighting plus 3-day 24 h food records method was validated in 2002 (23).

Doctors with special training conducted measurements of body weight (wearing light clothes), height (without shoes), and waist circumference following standardized protocols (24). Body mass index (BMI) was calculated as weight in kilograms divided by the square of height in meters. Cut points based on the Chinese criteria were 24 ≤ BMI <28 kg/m^2^ for overweight and BMI ≥28 kg/m^2^ for obesity (25); and the cut point of waist circumference was ≥80 cm in women for central obesity (26).

### Statistical analysis

Baseline characteristics by quartiles of daily BCAA intakes were compared using One-way ANOVA and chi-square tests. We calculated the odd ratios (ORs) and 95% confidential intervals (CIs) of general overweight and abdominal obesity by quartiles of daily BCAA intakes using a logistic regression. The analyses were first performed adjusting for age (Model 1), then for education, family history of diabetes, family income, current smoking, passive smoking, current alcohol drinking, leisure time physical activities, and sitting time (Model 2), and further for daily energy intake (Model 3). Restricted cubic spline analysis was used to examine the full-range association of daily BCAA intakes with obesity risk. Statistical analyses were performed using SPSS statistics V.25.0 (IBM) and SAS version 9.3 (SAS Institute Inc., Cary, NC, United States). Two-sided *p* < 0.05 was considered statistically significant.

## Results

Current alcohol drinking and daily energy intake were positively associated with daily BCAA intakes postpartum (Table 1).

**Table 1:** Baseline characteristics by dietary branched-chain amino acid intakes.

	Quartiles of branched-chain amino acids				*p* value
	1	2	3	4	
No. of participants	315	317	315	316	
Age, years	32.0 ± 3.4	32.4 ± 3.7	32.5 ± 3.4	32.6 ± 3.6	0.18
Baseline survey after delivery, months	27.3 ± 10.4	28.6 ± 11.5	26.9 ± 9.8	26.9 ± 10.2	0.14
Body mass index, kg/m^2^	24.6 ± 4.2	23.7 ± 3.7	23.9 ± 3.8	24.4 ± 4.0	0.01
Body mass index groups, %					0.13
< 24 kg/m^2^	50.5	59.0	54.9	51.6	
≥ 24 kg/m^2^	49.5	41.0	45.1	48.4	
Education, %					0.20
< 13 years	27.6	22.1	21.3	19	
≥ 13 to <16 years	66.7	69.7	71.4	72.5	
≥ 16 years	5.7	8.2	7.3	8.5	
Family income, %					0.79
< 5,000 yuan/month	27.6	30.0	24.8	27.5	
5,000 ~ 7,999 yuan/month	38.7	35.6	37.8	35.4	
≥ 8,000 yuan/month	33.7	34.4	37.5	37.0	
Current smoking, %	3.2	0.9	2.2	1.6	0.22
Passive smoking, %	52.4	52.7	54.3	56.0	0.79
Current alcohol, %	20.0	25.5	22.5	32.0	0.01
Leisure time physical activity, %					0.62
0 min/day	50	52.4	55.3	48.3	
1 ~ 29 min/day	48.7	44.9	42.4	49.2	
≥ 30 min/day	1.3	2.8	2.3	2.5	
Sitting time, h/day	3.21 ± 2.01	3.30 ± 2.33	3.03 ± 1.87	3.35 ± 2.16	0.02
Daily energy consumption, kcal/day	1,325 ± 320	1,587 ± 329	1779 ± 306	2078 ± 422	<0.001
Branched chain amino acids, mg/day	3,627 ± 913	5,592 ± 422	7,251 ± 495	10,491 ± 2,225	<0.001
Isoleucine	931 ± 236	1,421 ± 120	1841 ± 143	2,671 ± 569	<0.001
Leucine	1,674 ± 429	2,581 ± 203	3,337 ± 247	4,817 ± 1,015	<0.001
Valine	1,022 ± 270	1,590 ± 151	2073 ± 159	3,003 ± 659	<0.001

Values are means ± SDs, or %. Reproduced from Yang et al. (27) pre-print, licensed under CC BY 4.0.

**Figure 2:**
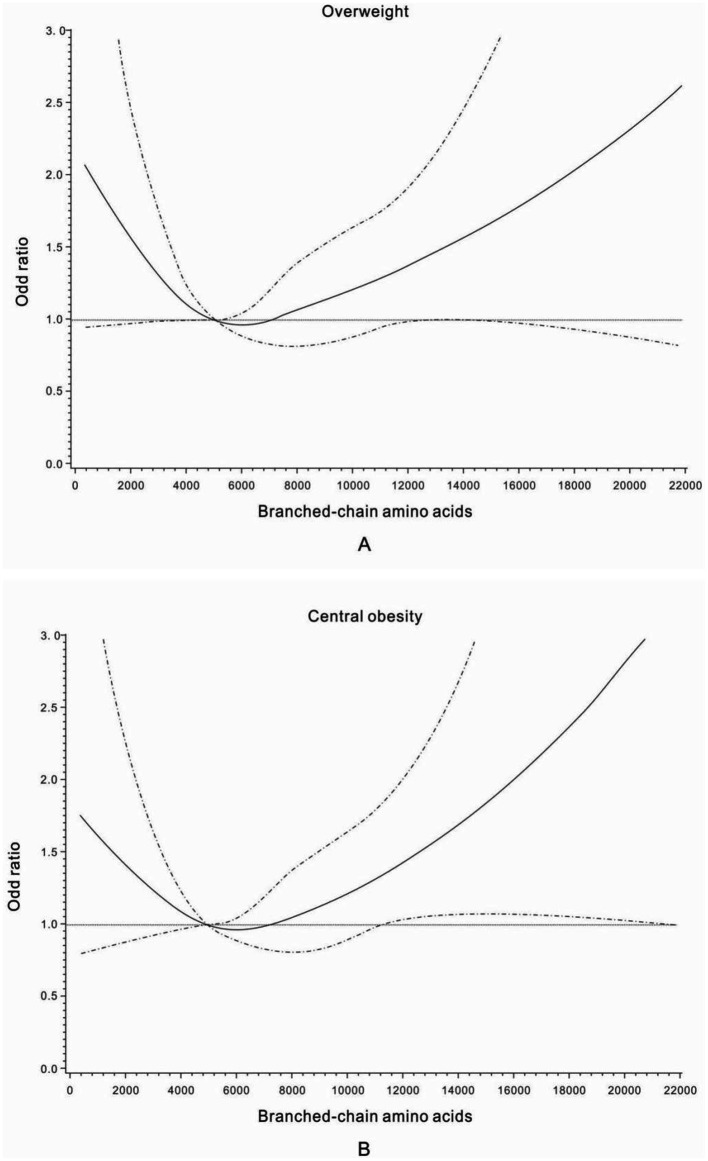
Odds ratios for general overweight **(A)** and central obesity **(B)** by different dietary intake levels of branched-chain amino acids (mg/day). Adjusted for age, education, family history of diabetes, family income, current smoking, passive smoking, current alcohol drinking, leisure time physical activities, and sitting time.

The U-shaped trend associations of dietary intakes of BCAAs, isoleucine, leucine, and valine with the general overweight risk were found, and a nadir of dietary intakes of BCAAs at Quartile 2 was chosen as the reference group in the analyses of overweight risk across quartiles of daily BCAA intakes (Table 2; Figure 2). The multivariable-adjusted (age, education, family income, family history of diabetes, current smoking, current alcohol drinking, leisure time physical activities, and sitting time – Model 2) odds ratios (ORs) for general overweight across quartiles of daily BCAA intakes were 1.42 (95% confidence interval [CI] 1.02–1.97), 1.00 (reference), 1.21 (95% CI 0.88–1.68), and 1.31 (95% CI 0.95–1.81), respectively. Women with the lowest or highest quartile of daily intakes of isoleucine and leucine had a significantly increased risk of general overweight compared with women at quartile 2 of daily isoleucine and leucine intakes (reference group) in the multivariable-adjusted analyses (Model 2). After further adjustment of daily energy intake, an increased risk of general overweight was only found among women with the lowest quartile of daily intakes of BCAAs (OR 1.49; 95% CI 1.06–2.09), isoleucine (OR 1.48; 95% CI 1.06–2.07), leucine (OR 1.51; 95% CI 1.08–2.11), and valine (OR 1.44; 95% CI 1.03–2.02) compared with women at quartile 2 of daily intakes of BCAAs, isoleucine, leucine, and valine (reference group), respectively.

**Table 2:** Odds ratios of overweight by branched chain amino acids categories.

	No. of participants	No. of cases	Odds ratio (95% confidence intervals)		
			Model 1	Model 2	Model 3
Branched chain amino acids					
Quartile 1	315	156	1.41 (1.03, 1.93)	1.42 (1.02, 1.97)	1.49 (1.06, 2.09)
Quartile 2	317	130	1	1	1
Quartile 3	315	142	1.18 (0.86, 1.62)	1.21 (0.88, 1.68)	1.17 (0.84, 1.63)
Quartile 4	316	153	1.35 (0.98, 1.84)	1.31 (0.95, 1.81)	1.20 (0.83, 1.72)
Isoleucine					
Quartile 1	315	155	1.37 (1.00, 1.87)	1.43 (1.03, 1.98)	1.48 (1.06, 2.07)
Quartile 2	316	131	1	1	1
Quartile 3	316	138	1.10 (0.80, 1.50)	1.15 (0.83, 1.59)	1.12 (0.80, 1.56)
Quartile 4	316	157	1.40 (1.02, 1.91)	1.40 (1.01, 1.93)	1.31 (0.91, 1.87)
Leucine					
Quartile 1	315	158	1.44 (1.05, 1.97)	1.45 (1.05, 2.01)	1.51 (1.08, 2.11)
Quartile 2	316	130	1	1	1
Quartile 3	316	134	1.06 (0.77, 1.45)	1.04 (0.75, 1.44)	1.01 (0.73, 1.41)
Quartile 4	316	159	1.45 (1.06, 1.99)	1.42 (1.03, 1.97)	1.32 (0.92, 1.90)
Valine					
Quartile 1	315	158	1.42 (1.04, 1.94)	1.38 (0.99, 1.91)	1.44 (1.03, 2.02)
Quartile 2	316	131	1	1	1
Quartile 3	316	138	1.10 (0.80, 1.51)	1.07 (0.77, 1.48)	1.03 (0.74, 1.43)
Quartile 4	316	154	1.35 (0.98, 1.84)	1.28 (0.93, 1.77)	1.17 (0.81, 1.68)

Model 1 adjusted for age; Model 2 adjusted for age, education, family history of diabetes, family income, current smoking, passive smoking, current alcohol drinking, leisure time physical activities, and sitting time; Model 3 adjusted for variables in Model 2 plus daily energy intake. Reproduced from Yang et al. (27) pre-print, licensed under CC BY 4.0.

The multivariable-adjusted (Model 2) U-shaped trend associations of daily intakes of BCAAs, isoleucine, leucine, and valine, with abdominal obesity risk (Table 3; Figure 2) were still observed among women with prior GDM. After further adjustment of daily energy intake, an increased risk of abdominal obesity was only found among women with the lowest quartile of daily intakes of BCAAs, isoleucine, leucine, and valine compared with women at quartile 2 of daily intakes of BCAAs, isoleucine, leucine, and valine (reference group), respectively.

**Table 3:** Odds ratios of central obesity by branched chain amino acids categories.

	No. of participants	No. of cases	Odds ratio (95% confidence intervals)		
			Model 1	Model 2	Model 3
**Branched chain amino acids**					
Quartile 1	315	166	1.38 (1.01, 1.88)	1.38 (0.99, 1.90)	1.50 (1.08, 2.11)
Quartile 2	317	142	1	1	1
Quartile 3	315	152	1.15 (0.84, 1.57)	1.19 (0.86, 1.64)	1.11 (0.80, 1.54)
Quartile 4	316	170	1.43 (1.05, 1.96)	1.43 (1.04, 1.98)	1.21 (0.84, 1.74)
**Isoleucine**	1,263	630			
Quartile 1	315	164	1.28 (0.94,1.76)	1.32 (0.95, 1.82)	1.42 (1.02, 1.99)
Quartile 2	316	145	1	1	1
Quartile 3	316	149	1.05 (0.77, 1.44)	1.12 (0.81, 1.54)	1.05 (0.76, 1.46)
Quartile 4	316	172	1.41 (1.03, 1.92)	1.44 (1.04, 1.99)	1.24 (0.86, 1.78)
**Leucine**	1,263	630			
Quartile 1	315	168	1.37 (1.00, 1.87)	1.36 (0.99, 1.88)	1.48 (1.06, 2.07)
Quartile 2	316	144	1	1	1
Quartile 3	316	143	0.98 (0.72, 1.35)	0.98 (0.71, 1.35)	0.92 (0.66, 1.28)
Quartile 4	316	175	1.48 (1.08, 2.03)	1.49 (1.08, 2.05)	1.26 (0.87, 1.80)
**Valine**	1,263	630			
Quartile 1	315	172	1.53 (1.12, 2.10)	1.46 (1.06, 2.02)	1.60 (1.15, 2.24)
Quartile 2	316	146	1	1	1
Quartile 3	316	142	1.19 (0.87, 1.63)	1.17 (0.85, 1.61)	1.08 (0.78, 1.50)
Quartile 4	316	170	1.48 (1.08, 2.02)	1.44 (1.04, 1.99)	1.19 (0.83, 1.71)

Model 1 adjusted for age; Model 2 adjusted for age, education, family history of diabetes, family income, current smoking, passive smoking, current alcohol drinking, leisure time physical activities, and sitting time; Model 3 adjusted for variables in Model 2 plus daily energy intake.

## Discussion

In this GDM women study, we found U-shaped trend associations of dietary intakes of BCAAs, isoleucine, leucine, and valine with general overweight and abdominal obesity risks among GDM women. Women with the lowest quartiles of daily intakes of BCAAs compared with women at quartile 2 of BCAA intakes had a significantly increased risk of obesity.

BCAAs belong to the essential amino acids that must be obtained from daily diet. The main sources of BCAAs from daily diet are high-protein foods such as dairy products, meat, eggs, as well as non-animal plant foods such as nuts and beans. Because of the positive physiological function and energy metabolism of BCAAs on tissues and cells *in vitro*, the supplementation of BCAAs is widely used for increasing muscle mass and enhancing fat loss among athletes for decades. Based on their widely use in daily life and important influence on metabolism, intensive investigations have focused on the potential role of BCAAs in the pathogenesis of obesity, insulin resistance and diabetes. A recent animal experiment from Ming L et al. showed that C57BL/6 J mice fed with restriction of BCAAs in high fat diet for 16 weeks maintained body weight, fat volume, and adipose tissue weight compared with the standard chow group, which prevented obesity and insulin resistance induced by high fat diet (28). Another animal experiment found similar results that specifically reducing BCAAs in diet promoted rapid fat weight loss and kept weight normalization without caloric restriction through transiently inducing fibroblast growth factor 21 (FGF21) and increasing energy expenditure (29).

Some population-based studies also investigated the associations between BCAAs and obesity in recent years. The “INTERMAP STUDY” is a cross-sectional epidemiological investigation including multi-ethnic populations from China, Japan, the US and UK with participants aged 40–59 years without diabetes. The results showed that higher dietary BCAA intakes were associated with lower prevalence of overweight and obesity (14). Another recent study from Iran showed a different conclusion (13). The study recruited 8,691 adults aged from 18 to 55 years old. The results showed that dietary BCAA intakes were associated with an increased odds of general obesity, especially among men, while no significant association was observed between dietary BCAAs and abdominal obesity. An increasing sedentary lifestyle and unhealthy dietary habits contribute to an increased obesity rate in China (30) especially among young people. A study from young adults in northern China showed that the dietary BCAA intake ratio is inversely associated with general overweight/obesity and abdominal obesity (15). However, some other studies had inconsistent results. A study in a middle-aged and elderly Chinese population aged 40–79 years showed a positive correlation between BCAA intakes and BMI and waist circumference (31). A recent study from our group showed that daily BCAA intakes were associated with increased risks for overweight and insulin resistance among the children of mothers with GDM, but this association was not fully independent of children’s daily energy intake (32).

Although some animal experiments and human studies mentioned above have investigated the relationship between BCAA intakes and body weight, the studies on the relationship between dietary BCAAs and obesity risk among GDM women are lacking. GDM is a serious pregnancy complication worldwide (1). Women with a history of GDM and postpartum obesity have an increased long-term diabetes risk (18, 33). Moreover, GDM women generally are overweight or obese after delivery (17), thus controlling body weight and reducing the obesity rate among GDM women postpartum are more important to reduce the long term risk of diabetes. A study among obese pregnant women showed that women who developed GDM had higher blood BCAA levels compared with women without GDM (34). Recent research from the CARDIA cohort study indicated that BCAA intakes were associated with increased odds of GDM (35). To our knowledge, there are no previous studies investigating the associations of BCAA intakes with overweight and abdominal obesity among GDM women postpartum. The present study found that lower intakes of BCAAs might increase overweight and abdominal obesity risks among women with a GDM history in the period of postpartum. The results were not consistent with previous studies, the reason may be due to the specially enrolled population in the present study. The present study may also provide a new way of dietary adjustment to avoid overweight and central obesity among women with a GDM history, for whom controlling weight is more important.

There were some strengths in the present study. First, this study was a large epidemiological study of GDM women in Tianjin, China. Second, the quantity of BCAA intakes was measured by a 3-day 24 h food record that was different from previous studies and more practical for adjustment of the daily diet structure (36). Third, as far as we know, many investigations focused on the relation of dietary BCAA intakes with obesity risk in the general population. This is the first study aiming at women with prior GDM in a short postpartum period. There were also some limitations in our study. First, the cross-sectional study design did not allow us to look at temporal relationships. Second, the present study only aimed at women with GDM in China. It could not represent the general population. Therefore, future studies are needed to verify the situation in other larger regions worldwide. Third, the measurement of BCAA intakes in our study was only conducted in a short term, which may be less representative of individuals’ long term dietary patterns, and may attenuate the associations between dietary BCAA intakes and obesity risk (37). Individual dietary habits are influenced by a host of social, cultural, customary and economic factors, thus assessments.

of diet in a relatively homogeneous population may weaken the association between diet and the disease. Last, GDM in the present study was diagnosed as either diabetes or impaired glucose tolerance using the 1999 WHO criteria. Although women with pre-pregnancy diabetes were excluded, our study might include some women with diabetes in pregnancy using the new GDM diagnosis criteria.

## Conclusion

The present study found that lower intakes of dietary intakes of BCAAs, isoleucine, leucine, and valine were significantly associated with increased risks of general overweight and abdominal obesity among GDM women. Only moderate intakes of BACCs were beneficial for weight control among GDM women during the postpartum period. More studies are needed to confirm this finding.

## Data Availability

The raw data supporting the conclusions of this article will be made available by the authors, without undue reservation.
